# Oligodendrocyte ferroptosis: Novel mechanisms in multiple sclerosis

**DOI:** 10.4103/NRR.NRR-D-25-01018

**Published:** 2025-11-25

**Authors:** Valentina Saverio, Dmitry Lim, Marco Corazzari

**Affiliations:** Department of Health Sciences, School of Medicine, Center for Translational Research on Autoimmune and Allergic Disease (CAAD), University of Piemonte Orientale, Novara, Italy; Department of Pharmaceutical Sciences, University of Piemonte Orientale, Novara, Italy

Myelination in the central nervous system (CNS) is a highly intricate process, exclusive to vertebrates, that relies on the coordinated interaction between oligodendrocytes (OLs) and neurons. In addition to their role in forming myelin, accumulating evidence indicates that OLs provide crucial trophic support to axons, contributing to normal CNS function. Notably, OL injury and loss are observed in a variety of human conditions, including stroke, traumatic injuries of the brain and spinal cord, as well as neurodegenerative disorders such as multiple sclerosis (MS).

It is well recognized that the early loss of myelin-producing oligodendrocytes constitutes a primary event contributing to neuronal degeneration and the subsequent pathological progression of MS. In this disease, myelin-producing oligodendrocytes became compromised, resulting in the disruption of the myelin sheath that normally insulates neuronal axons, a process referred to as demyelination, although the exact cause of myelin-producing oligodendrocytes demise in MS remains largely unclear. Furthermore, the modest efficacy of current treatments for progressive MS highlights the urgent need for alternative therapeutic strategies, including those aimed at neuroprotection and promoting functional recovery.

Recently, a new form of cell death called ferroptosis has been described, which may play a role in OL loss during the onset and progression of MS (Stockwell, 2022). Ferroptosis is genetically and morphologically distinct from other major forms of cell death, including apoptosis, necrosis, and autophagic cell death, and is characterized by the production of reactive lipid species (lipid-reactive oxygen species [ROS]), which are regarded as the primary executioners of the process. The accumulation of lipid-ROS arises from an increased intracellular labile iron pool and/or heightened activity of iron-dependent enzymes such as lipoxygenases. This is accompanied by a diminished cellular capacity to detoxify lipid-ROS, often due to reduced expression or activity of key antioxidant enzymes, most notably glutathione peroxidase 4 (Gagliardi et al., 2020).

**Emerging connections linking ferroptosis and multiple sclerosis:** Over the past decade, multiple studies have reported decreased glutathione peroxidase 4 expression alongside the accumulation of the lipid peroxidation byproduct 4-HNE in the gray matter of MS patients, as well as in the spinal cord of experimental autoimmune encephalomyelitis (EAE) mice, a widely used and well-established animal model of MS (Hu et al., 2018; Nguyen et al., 2025). Moreover, the presence of abnormal mitochondrial morphology in neurons — characterized by disrupted outer membranes and reduced cristae — alongside well-documented alterations in iron distribution within MS lesions, as confirmed by magnetic resonance imaging, histopathological analysis, and X-ray fluorescence imaging, strongly suggests the involvement of ferroptosis. Supporting this hypothesis, the use of deferoxamine, an iron chelator and ferroptosis inhibitor, has been shown to attenuate disease progression in EAE mice.

Indeed, in normal-appearing white matter, oligodendrocyte iron content progressively declines due to impaired uptake and enhanced export, potentially driven by inflammatory signaling. Conversely, in active lesions, oligodendrocyte death results in iron release into the extracellular space, where it accumulates in microglia and macrophages and amplifies oxidative damage. Inflammatory cytokines such as tumor necrosis factor alpha (TNF-α) and interferon-γ (IFN-γ) further exacerbate iron dysregulation, increasing oligodendrocyte vulnerability to ferroptosis. This imbalance not only contributes to neurodegeneration but also impedes remyelination by depriving oligodendrocyte precursor cells of the iron necessary for differentiation and myelin synthesis (reviewed in Ye et al., 2024).

Ferroptosis has been shown to underlie the rapid demyelination triggered by cuprizone (CZ), a commonly used copper chelator. CZ specifically triggers ferroptosis in oligodendrocytes, leading to their rapid loss and subsequent acute demyelination, thereby inducing a pathological phenotype resembling multiple sclerosis (Jhelum et al., 2020). Although cuprizone is widely employed to study experimental approaches aimed at preventing myelin loss or enhancing remyelination, the exact mechanisms through which copper chelation induces oligodendrocyte death are not yet fully understood. Notably, CZ promotes the mobilization of bioactive iron via NCOA4-mediated ferritinophagy, while concurrently increasing the expression of transferrin receptor 1, thereby enhancing iron uptake and subsequent lipid ROS generation. Furthermore, copper-dependent enzymes, especially ferroxidases, contribute to iron-driven membrane oxidation, culminating in ferroptotic cell death.

**Oligodendrocyte maturation and ferroptosis intersection:** OLs represent the primary iron-rich cells within the brain. Iron is crucial for the proper activity of mitochondrial respiratory chain complexes. Numerous enzymes involved in adenosine triphosphate generation, including HMG-CoA reductase and NADH dehydrogenase, are iron-dependent and are particularly enriched in OLs relative to other CNS cell types. In addition, OLs exhibit the highest metabolic activity in the brain due to the extensive lipid synthesis required for myelin production. As a result, these cells have elevated adenosine triphosphate demands and consume substantial amounts of oxygen. Combined with their relatively low glutathione levels, the high respiration rate and the iron dependency possibly explain the vulnerability of OLs to both oxidative stress and cell death.

Oligodendrocyte maturation requires the biosynthesis of cholesterol and fatty acids to form myelin, a process that depends on iron-dependent enzymes such as glucose-6-phosphate dehydrogenase. With aging, iron progressively accumulates, and disruptions in its homeostasis can undermine OL function and viability, ultimately contributing to demyelination and neurodegeneration. Elucidating the mechanisms that govern OL survival and death is therefore essential for strategies aimed at preserving myelin and protecting axons, particularly in conditions such as MS. Emerging evidence indicates that OLs are particularly vulnerable to iron-induced oxidative stress, which can drive ferroptotic cell death.

Recently, it has been reported that a human OL precursor–derived cell line is sensitive to ferroptosis execution triggered by erastin, a well-known and widely used ferroptosis inducer. Notably, upon maturation, these cells became resistant to erastin, which was associated with increased expression of SLC7A11. This transporter mediates cystine uptake, supporting subsequent glutathione synthesis. Glutathione is then utilized by glutathione peroxidase 4 to neutralize lipid-derived ROS, thereby preventing the execution of ferroptosis (Hoshino et al., 2020).

Recent evidence indicates that mature oligodendrocytes exhibit resistance to ferroptotic cell death irrespective of the initiating trigger, implying that mechanisms beyond SLC7A11 upregulation contribute to this protection (Saverio et al., 2025).

Indeed, increased expression of AKR1C1 was observed in mature, ferroptosis resistant OLs, and its inhibition or down-regulation consistently re-sensitized these cells to ferroptosis execution. AKR1C1, a member of aldo-keto reductase superfamily, is a NADPH-dependent enzyme that reduces aldehydes and ketones to alcohols, reducing lipid peroxides (Gagliardi et al., 2019). This observation is consistent with AKR1C1 being the predominant AKR isoform in the CNS, which may account for its significant role in limiting the susceptibility of mature OLs to ferroptotic cell death under physiological conditions.

Interestingly, evidence also indicates that nuclear factor erythroid 2-related factor 2 (NRF2) contributes to the upregulation of AKR1C1 in mature OLs, as pharmacological inhibition of NRF2 abolishes the differentiation-associated increase in AKR1C1 expression. Taken together, these observations support a model in which, during OLs differentiation, elevated ROS activate NRF2, which in turn promotes AKR1C1 expression. This mechanism likely contributes to the detoxification of lipid-ROS and underlies the resistance of mature OLs to ferroptosis under physiological conditions.

**Inflammatory landscape of multiple sclerosis:** **a role of ferroptosis?** Focal inflammatory lesions with disruption of the blood–brain barrier, which allows the infiltration of T and B lymphocytes, leading to myelin disruption, are a hallmark of MS onset. Indeed, recruited monocytes, activated microglia, and infiltrating macrophages release substantial levels of proinflammatory molecules and ROS. The intrinsically low ability to cope with oxidative stress — characteristic of CNS inflammation at MS lesion sites — may impair the physiological ability of oligodendrocyte precursor cells to sustain OLs differentiation and remyelination. Additionally, oxidized lipids, hallmarks of ferroptosis, are observed in the brains of individuals with MS, and their localization within lesions suggests that ferroptotic mechanisms may contribute to mature oligodendrocyte loss. Interestingly and in line with the above hypothesis, the early induction of ferroptosis in the CNS and spinal cord of the EAE mouse model of MS has been recently reported (Luoqian et al., 2022).

It is also important to note that ferroptosis execution releases active factors (damage-associated molecular patterns) that have been proven to promote T-cell activation and stimulate cytokine production by Th1 and Th17 cells, which ultimately might lead to neuron death. Cytokines released by autoreactive lymphocytes are thought to enhance immune activity, thereby intensifying myelin damage and contributing to OL loss. Among these mediators, proinflammatory molecules such as IFN-γ and TNF-α have been strongly linked to MS pathogenesis. Elevated levels of IFN-γ have been observed in both the serum and brain lesions of MS patients, while experimental models demonstrate that heightened TNF-α within the CNS can drive demyelination. Furthermore, these cytokines can trigger apoptotic death of human OLs in a dose-dependent manner (Buntinx et al., 2004).

In the EAE model, ferroptotic cell death has been shown to actively drive adaptive immune activation. Mechanistically, ferroptosis in oligodendrocytes and CNS-resident cells leads to the release of damage-associated molecular patterns, including oxidized lipids and other oxidative by-products, which engage pattern-recognition receptors on antigen-presenting cells. This interaction enhances antigen-presenting cell maturation and antigen presentation, promoting T-cell receptor signaling and clonal expansion of autoreactive T cells. Pharmacological inhibition or genetic suppression of ferroptosis (e.g., ACSL4 knockdown) markedly reduces damage-associated molecular pattern release, T-cell activation, and neuroinflammatory damage, demonstrating a direct causal link between ferroptosis and adaptive immune responses relevant to MS pathology (Luoqian et al., 2022).

Interestingly, combined TNF-α and IFN-γ show a synergic effect in the induction of ferroptotic cell death in mature OLs through a consistent downregulation of AKR1C1 (Saverio et al., 2025). The identification of AKR1C1 as a natural protective protein toward ferroptotic cell death execution and the mechanism at the basis of its deregulation during MS-associated neuroinflammation indubitably represents an important step forward to better and deeply understand the pathogenesis of this highly invalidating disease.

**Regulatory roles of microRNA in ferroptosis — implication for multiple sclerosis:** Dysregulated expression of specific microRNAs has been linked to multiple neurodegenerative disorders, including Parkinson’s disease and Alzheimer’s disease. These small non-coding RNAs regulate a wide range of physiological and pathological processes within the nervous system, such as neuronal differentiation, synapse formation, and synaptic plasticity. Their contribution to neurological disease mechanisms is now receiving growing attention.

**Figure 1:**
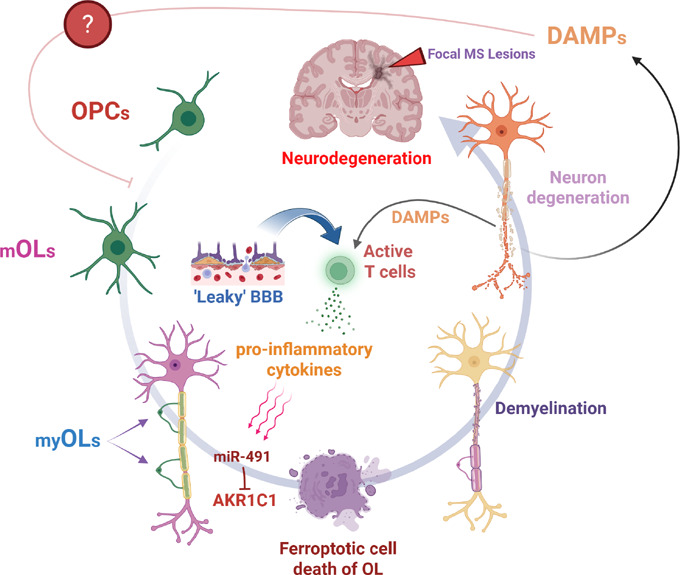
Oligodendrocyte demise during multiple sclerosis (MS) onset and progression. Schematic representation of the circuit summarizing oligodendrocyte differentiation and death in MS: mature oligodendrocytes (mOLs) arise from the differentiation of stem-like cells called oligodendrocyte precursor cells (OPCs). Terminally differentiated oligodendrocytes (myelinating OLs and myelin-producing oligodendrocytes [myOLs]) produce myelin sheaths that wrap around the axonal core of neurons, thereby electrically insulating segments of the axon. During MS onset and progression, a dysregulated blood–brain barrier (BBB) (“leaky” BBB) permits infiltration of activated T cells into the central nervous system. This leads to the release of proinflammatory cytokines, which downregulate the expression of the anti-ferroptotic factor AKR1C1 in OLs by upregulating miR-491. The resulting ferroptotic cell death of OLs causes demyelination, oligodendrocyte loss, and neuronal degeneration, ultimately forming the characteristic “focal MS lesions.” Additionally, the release of cellular components, such as damage-associated molecular patterns (DAMPs), may inhibit further OPC differentiation and replacement of dead OLs while simultaneously promoting T cell activation, thereby contributing to disease progression. Created with BioRender.com.

Analyses of brain tissues from MS patients have identified altered levels of multiple miRNAs, including miR-338, miR-155, and miR-491, which were elevated in white matter samples (Noorbakhsh et al., 2011). Among them, miR-491 has been linked to the regulation of AKR1C1. Experimental data indicate that forced expression of miR-491 *in vitro* reduces AKR1C1 levels, thereby increasing OL vulnerability to ferroptosis (Saverio et al., 2025). These observations suggest that activation of the ROS/NRF2 pathway supports AKR1C1 expression and confers resistance to ferroptotic death, whereas inflammation-driven induction of miR-491 in MS may compromise this protective mechanism (**Figure 1**).

**Conclusions:** Although the precise role of ferroptosis in MS pathogenesis remains to be fully elucidated, increasing evidence supports its involvement in oligodendrocyte degeneration, leading to demyelination and neuronal loss. Recent findings highlight AKR1C1 as a potential protective factor, suggesting that therapeutic strategies aimed at sustaining its expression in mature oligodendrocytes could mitigate inflammation-driven cell death and neurodegeneration in MS.

To further enhance the translational impact of these findings, future investigations should integrate cutting-edge approaches, such as spatiotemporal multi-omics technologies. These advanced methodologies offer the unique potential to dissect complex and dynamic molecular landscapes, including ferroptosis pathways, immune responses, and regenerative mechanisms, directly within defined cellular niches of MS lesions. Such integrative analyses, combined with validation in animal models and MS patient samples, will be crucial for confirming our in vitro results and accelerating the development of targeted therapeutic interventions.
